# Epidemiology of severe infections in Latin American intensive care units

**DOI:** 10.5935/0103-507X.20160051

**Published:** 2016

**Authors:** Guillermo Ortiz Ruiz, Carmelo Dueñas Castell

**Affiliations:** 1 Posgrado de Medicina Interna y Neumología, Universidad el Bosque - Bogotá, Colombia.; 2 Cuidado Critico, Hospital Santa Clara - Bogotá, Colombia.; 3 Universidad de Cartagena - Cartagena, Colombia.; 4 Unidad de Cuidados Intensivos, Gestión Salud, Linde HealthCare - Bogotá, Colombia.

Severe sepsis and septic shock are important causes of morbidity and mortality in patients admitted to intensive care units (ICU).^(1)^ These conditions are generally associated with multiple organ failure as a final outcome.^(1-4)^ Over the past 30 years, the worldwide incidence of sepsis has increased by 13.7% per year.^(1-4)^ It is therefore estimated that more than 18 million people suffer from sepsis each year, and more than five million of them die.^(1-4)^ This increase is arguably due to the increasing numbers of people aged over 65 years (60% of septic patients are more than 65 years old), more frequent diseases and therapies causing immunosuppression, and the widespread use of diagnostic and/or therapeutic invasive procedures.

Recent studies from Europe, the United Kingdom, Australia, and New Zealand have shown the incidence rates of sepsis and its mortality in ICUs.^(5-9)^ North American studies are limited to data obtained in four Canadian ICUs that participated in a multinational study and to data taken from administrative databases.^(1)^ These data reveal an incidence of sepsis in the ICU ranging from 11.8% to 37.4%, with mortality rates between 35% and 53.6% (both in the hospital and after 30 days).

## Latin American perspective

The majority of the representative epidemiologic reports of sepsis are from developed countries; in Latin America, the clinical and epidemiological approaches to the problem have sometimes been inappropriate in terms of research design, study population, and clinical outcomes.^(10)^ Cities are expanding rapidly in middle-income countries, but their supply of acute care services is unknown. Hospital bed per disease burden has been associated with gross domestic product, but ICU supply has not. Given that there are no well-recognized metrics for acute care services supply, it is not surprising that cities lack comprehensive data.^(11)^ In a convenience sample of 13 ICUs from low- and middle-income countries, specialty-trained staff and standardized processes of care such as checklists were frequently missing.^(12)^

It is unlikely that in Latin America there is a lower incidence of sepsis or a better prognosis for the condition than there is in the developed countries of the world. In EPISEPSIS Colombia,^(13)^ a prospective study that addressed the current status of sepsis in adult patients hospitalized in institutions of the highest level within the Colombian health system, we found that the frequency of severe sepsis and septic shock are far beyond the figures reported throughout the world. Although the mortality rates of patients who met the criteria for severe sepsis and septic shock (22% and 46%, respectively) are similar to those reported in other studies,^(1,14)^ the overall 28-day mortality rate of 19% is higher than expected, according to a mean Acute Physiologic and Chronic Health Evaluation II score of 11.5 (14%). In a multicenter observational cohort study, Silva concluded that sepsis is a major public health problem in Brazilian ICUs, with an incidence density of approximately 57 per 1000 patient-days. Moreover, there was a close association between American College of Chest Physicians/Society of Critical Care Medicine (ACCP/SCCM) categories and mortality rate.^(15)^

This intended evaluation of the global burden of sepsis turned out to be limited because of missing reliable population-based data from low- and middle-income countries. The true incidence and burden of sepsis in these countries remains uncertain because of a lack of information on the epidemiology of sepsis. Given the considerably higher prevalence of acute infections that may lead to sepsis in low- and middle-income countries where studies on the epidemiology of sepsis are missing, any estimates derived from high-income countries that add hospital-acquired to community-acquired cases may underestimate the true global cumulative incidence of sepsis. We think that in Latin America there is an interesting scenario for quality-improvement initiatives. In Latin American countries, there is a gap between scientific evidence and bedside care; such a gap is mainly explained by a lack of adequate workflow prioritizing timely access to care for patients with severe sepsis within hospitals, resistance to following guidelines, and lack of knowledge of staff members.^(13)^

Cost-effectiveness is also an important concern in the treatment of sepsis.^(1)^ A few studies indicated that the implementation of bundles is a cost-effective initiative, but the heterogeneity of scenarios, methods, and results does not allow for a definitive conclusion.^(16)^ Thus, it would be very relevant to demonstrate cost-effectiveness in the context of an emerging economy. In a recent study from Brazil,^(17)^ the main finding was that a multifaceted intervention in a vertically integrated system of private hospitals in an emerging country was capable of achieving very high compliance with the surviving sepsis campaign (SSC) resuscitation bundle during 7 trimesters of follow-up. Using an adjusted analysis, they were able to demonstrate that both compliance and the length of time of the intervention were associated with a reduction in the mortality rate. This program could also reduce the cost of care for septic patients.

## What about the future?

In Latin America, we need to improve diagnostic measures and increase awareness, and we need more accurate International Classification of Diseases (ICD) coding for sepsis and severe sepsis to know the real incidence and lethality of this condition in our region. If we can have a more accurate view of what is actually happening in our region, we can evaluate the impact and cost of implementing management bundles.^(17)^ We must improve our knowledge about our local epidemiology and bacterial resistance patterns to improve the therapeutic approach to sepsis and to evaluate preventive strategies. Another significant challenge is to address outbreaks of particular interest in our region, such as the Zika virus. The Zika outbreak began in April 2015 in Brazil and subsequently spread to other countries in South America, Central America, and the Caribbean. In January 2016, the World Health Organization (WHO) said that the virus was likely to spread throughout most of the Americas by the end of the year;^(18)^ and in February 2016, the WHO declared that the cluster of microcephaly and Guillain-Barré syndrome cases reported in Brazil - strongly suspected to be associated with the Zika virus outbreak- was a public health emergency of international concern.^(18)^ The only way to face these epidemics is with a very well structured health system in the region, associated with advanced developments in technology and information systems. Critical situations may aid policy makers and researchers regarding decisions on appropriate investments in infrastructure required to treat the acute disease in the intensive care setting. In emerging countries, organizational factors, including the implementation of protocols, are potential targets to improve patient outcomes and resource use in ICUs.^(19)^

The PIRO (predisposition, insult, response, and organ dysfunction) model has been used as a conceptual framework for the understanding of sepsis and can offer great advantages when setting clinical and research goals or targets in Latin America.^(20)^ In table 1, we use the PIRO model to describe some aspects that need to be improved for understanding sepsis in our region.

**Table 1:** Future of sepsis research in Latin America

Patients	Infection	Response	Organ failure
Determine if there are specific demographic characteristics or comorbidities in Latin America and if there are differences in the region.	Establish the most common germs and patterns of multidrug resistance.	To evaluate the usefulness of biomarkers as presepsina and procalcitonin.	Determine whether there are phenotypes of sepsis in Latin America and risk factors associated with specific organ dysfunction
